# Supplementary material for: COZOID: contact zone identifier for visual analysis of protein-protein interactions
Source: BMC Bioinformatics. 2018 Apr 6;19:125. doi: 10.1186/s12859-018-2113-6 (PMC5889581; doi:10.1186/s12859-018-2113-6)
Supplement: Supplementary file 2 — Software build. Executable binary file of the software tool. (ZIP 118,784 kb) [file 12859_2018_2113_MOESM2_ESM.zip › cozoid/platform/docs/testng-6.5.1-javadoc.zip!/org/testng/SuiteRunner.html]

SuiteRunner (TestNG)


---


|  |  |  |
| --- | --- | --- |
| **Overview** \| **Package** \| **Class** \| **Tree** \| **Deprecated** \| **Index** \| **Help** | |  |
| **PREV CLASS**   **NEXT CLASS** | **FRAMES**    **NO FRAMES**     **All Classes** |  |
| SUMMARY: NESTED \| FIELD \| CONSTR \| METHOD | DETAIL: FIELD \| CONSTR \| METHOD |  |


---


## org.testng Class SuiteRunner

```
java.lang.Object
  org.testng.SuiteRunner
```

**All Implemented Interfaces:**: java.io.Serializable, IAttributes, IInvokedMethodListener, ISuite, ITestNGListener

---

``` public class SuiteRunner extends java.lang.Object implements ISuite, java.io.Serializable, IInvokedMethodListener ```

`SuiteRunner` is responsible for running all the tests included in one
suite. The test start is triggered by `run()` method.

**Author:**
:   Cedric Beust, Apr 26, 2004

**See Also:**: Serialized Form

---

| **Constructor Summary** | |
| --- | --- |
|  | `SuiteRunner(org.testng.internal.IConfiguration configuration, XmlSuite suite, java.lang.String outputDir)` |
|  | `SuiteRunner(org.testng.internal.IConfiguration configuration, XmlSuite suite, java.lang.String outputDir, ITestRunnerFactory runnerFactory)` |
|  | `SuiteRunner(org.testng.internal.IConfiguration configuration, XmlSuite suite, java.lang.String outputDir, ITestRunnerFactory runnerFactory, boolean useDefaultListeners)` |
| `protected` | `SuiteRunner(org.testng.internal.IConfiguration configuration, XmlSuite suite, java.lang.String outputDir, ITestRunnerFactory runnerFactory, boolean useDefaultListeners, IMethodInterceptor methodInterceptor, java.util.List<IInvokedMethodListener> invokedMethodListeners, java.util.List<ITestListener> testListeners)` |


| **Method Summary** | |
| --- | --- |
| `protected  void` | `addListener(ISuiteListener reporter)`             Registers ISuiteListeners interested in reporting the result of the current suite. |
| `void` | `addListener(ITestNGListener listener)` |
| `void` | `afterInvocation(IInvokedMethod method, ITestResult testResult)` |
| `void` | `beforeInvocation(IInvokedMethod method, ITestResult testResult)` |
| `java.util.List<IInvokedMethod>` | `getAllInvokedMethods()` |
| `java.util.List<ITestNGMethod>` | `getAllMethods()` |
| `org.testng.internal.annotations.IAnnotationFinder` | `getAnnotationFinder()`             Returns the annotation finder for the given annotation type. |
| `java.lang.Object` | `getAttribute(java.lang.String name)` |
| `java.util.Set<java.lang.String>` | `getAttributeNames()` |
| `java.util.Collection<ITestNGMethod>` | `getExcludedMethods()` |
| `java.lang.String` | `getHost()` |
| `java.util.Collection<ITestNGMethod>` | `getInvokedMethods()`             Retrieves the list of all the methods that were invoked during this run. |
| `java.util.Map<java.lang.String,java.util.Collection<ITestNGMethod>>` | `getMethodsByGroups()`             Retrieves the map of groups and their associated test methods. |
| `java.lang.String` | `getName()` |
| `IObjectFactory` | `getObjectFactory()` |
| `IObjectFactory2` | `getObjectFactory2()` |
| `java.lang.String` | `getOutputDirectory()` |
| `java.lang.String` | `getParallel()` |
| `java.lang.String` | `getParameter(java.lang.String parameterName)`             FIXME: should be removed? |
| `java.util.List<IReporter>` | `getReporters()` |
| `java.util.Map<java.lang.String,ISuiteResult>` | `getResults()` |
| `SuiteRunState` | `getSuiteState()`             Retrieves the shared state for a suite. |
| `XmlSuite` | `getXmlSuite()` |
| `static void` | `ppp(java.lang.String s)` |
| `java.lang.Object` | `removeAttribute(java.lang.String name)`             Remove the attribute |
| `void` | `run()`             Triggers the start of running tests included in the suite. |
| `void` | `setAttribute(java.lang.String name, java.lang.Object value)`             Set a custom attribute. |
| `void` | `setHost(java.lang.String host)` |
| `void` | `setObjectFactory(ITestObjectFactory objectFactory)` |
| `void` | `setReportResults(boolean reportResults)` |
| `void` | `setSkipFailedInvocationCounts(java.lang.Boolean skipFailedInvocationCounts)` |

**Methods inherited from class java.lang.Object**

`clone, equals, finalize, getClass, hashCode, notify, notifyAll, toString, wait, wait, wait`

**Constructor Detail**

### SuiteRunner

```
public SuiteRunner(org.testng.internal.IConfiguration configuration,
                   XmlSuite suite,
                   java.lang.String outputDir)
```

---


### SuiteRunner

```
public SuiteRunner(org.testng.internal.IConfiguration configuration,
                   XmlSuite suite,
                   java.lang.String outputDir,
                   ITestRunnerFactory runnerFactory)
```

---


### SuiteRunner

```
public SuiteRunner(org.testng.internal.IConfiguration configuration,
                   XmlSuite suite,
                   java.lang.String outputDir,
                   ITestRunnerFactory runnerFactory,
                   boolean useDefaultListeners)
```

---


### SuiteRunner

```
protected SuiteRunner(org.testng.internal.IConfiguration configuration,
                      XmlSuite suite,
                      java.lang.String outputDir,
                      ITestRunnerFactory runnerFactory,
                      boolean useDefaultListeners,
                      IMethodInterceptor methodInterceptor,
                      java.util.List<IInvokedMethodListener> invokedMethodListeners,
                      java.util.List<ITestListener> testListeners)
```


**Method Detail**

### getXmlSuite

```
public XmlSuite getXmlSuite()
```

:   **Specified by:**: `getXmlSuite` in interface `ISuite`
:   **Returns:**: The representation of the current XML suite file.

---


### getName

```
public java.lang.String getName()
```

:   **Specified by:**: `getName` in interface `ISuite`
:   **Returns:**: the name of this suite.

---


### setObjectFactory

```
public void setObjectFactory(ITestObjectFactory objectFactory)
```

---


### setReportResults

```
public void setReportResults(boolean reportResults)
```

---


### getParallel

```
public java.lang.String getParallel()
```

:   **Specified by:**: `getParallel` in interface `ISuite`
:   **Returns:**: true if the tests must be run in parallel.

---


### run

```
public void run()
```

:   **Description copied from interface: `ISuite`**
:   Triggers the start of running tests included in the suite.

    :   **Specified by:**: `run` in interface `ISuite`

---


### getReporters

```
public java.util.List<IReporter> getReporters()
```

---


### addListener

```
protected void addListener(ISuiteListener reporter)
```

:   Registers ISuiteListeners interested in reporting the result of the current
    suite.

    :   **Parameters:**: `reporter` -

---


### addListener

```
public void addListener(ITestNGListener listener)
```

:   **Specified by:**: `addListener` in interface `ISuite`

---


### getOutputDirectory

```
public java.lang.String getOutputDirectory()
```

:   **Specified by:**: `getOutputDirectory` in interface `ISuite`
:   **Returns:**: The output directory used for the reports.

---


### getResults

```
public java.util.Map<java.lang.String,ISuiteResult> getResults()
```

:   **Specified by:**: `getResults` in interface `ISuite`
:   **Returns:**: The results for this suite.

---


### getParameter

```
public java.lang.String getParameter(java.lang.String parameterName)
```

:   FIXME: should be removed?

    :   **Specified by:**: `getParameter` in interface `ISuite`
    :   **Returns:**: The value of this parameter, or null if none was specified. **See Also:**: `ISuite.getParameter(java.lang.String)`

---


### getMethodsByGroups

```
public java.util.Map<java.lang.String,java.util.Collection<ITestNGMethod>> getMethodsByGroups()
```

:   **Description copied from interface: `ISuite`**
:   Retrieves the map of groups and their associated test methods.

    :   **Specified by:**: `getMethodsByGroups` in interface `ISuite`
    :   **Returns:**: A map where the key is the group and the value is a list of methods used by this group. **See Also:**: `ISuite.getMethodsByGroups()`

---


### getInvokedMethods

```
public java.util.Collection<ITestNGMethod> getInvokedMethods()
```

:   **Description copied from interface: `ISuite`**
:   Retrieves the list of all the methods that were invoked during this run.

    :   **Specified by:**: `getInvokedMethods` in interface `ISuite`
    :   **Returns:**: a collection of ITestNGMethods belonging to all tests included in the suite. **See Also:**: `ISuite.getInvokedMethods()`

---


### getExcludedMethods

```
public java.util.Collection<ITestNGMethod> getExcludedMethods()
```

:   **Specified by:**: `getExcludedMethods` in interface `ISuite`
:   **Returns:**: All the methods that were not included in this test run. **See Also:**: `ISuite.getExcludedMethods()`

---


### getObjectFactory

```
public IObjectFactory getObjectFactory()
```

:   **Specified by:**: `getObjectFactory` in interface `ISuite`
:   **Returns:**: The object factory used to create all test instances.

---


### getObjectFactory2

```
public IObjectFactory2 getObjectFactory2()
```

:   **Specified by:**: `getObjectFactory2` in interface `ISuite`

---


### getAnnotationFinder

```
public org.testng.internal.annotations.IAnnotationFinder getAnnotationFinder()
```

:   Returns the annotation finder for the given annotation type.

    :   **Specified by:**: `getAnnotationFinder` in interface `ISuite`
    :   **Returns:**: the annotation finder for the given annotation type.

---


### ppp

```
public static void ppp(java.lang.String s)
```

---


### setHost

```
public void setHost(java.lang.String host)
```

---


### getHost

```
public java.lang.String getHost()
```

:   **Specified by:**: `getHost` in interface `ISuite`
:   **Returns:**: The host where this suite was run, or null if it was run locally. The returned string has the form: host:port

---


### getSuiteState

```
public SuiteRunState getSuiteState()
```

:   **Description copied from interface: `ISuite`**
:   Retrieves the shared state for a suite.

    :   **Specified by:**: `getSuiteState` in interface `ISuite`
    :   **Returns:**: the share state of the current suite. **See Also:**: `ISuite.getSuiteState()`

---


### setSkipFailedInvocationCounts

```
public void setSkipFailedInvocationCounts(java.lang.Boolean skipFailedInvocationCounts)
```

---


### getAttribute

```
public java.lang.Object getAttribute(java.lang.String name)
```

:   **Specified by:**: `getAttribute` in interface `IAttributes`
:   **Parameters:**: `name` - The name of the attribute to return

---


### setAttribute

```
public void setAttribute(java.lang.String name,
                         java.lang.Object value)
```

:   **Description copied from interface: `IAttributes`**
:   Set a custom attribute.

    :   **Specified by:**: `setAttribute` in interface `IAttributes`

---


### getAttributeNames

```
public java.util.Set<java.lang.String> getAttributeNames()
```

:   **Specified by:**: `getAttributeNames` in interface `IAttributes`
:   **Returns:**: all the attributes names.

---


### removeAttribute

```
public java.lang.Object removeAttribute(java.lang.String name)
```

:   **Description copied from interface: `IAttributes`**
:   Remove the attribute

    :   **Specified by:**: `removeAttribute` in interface `IAttributes`
    :   **Returns:**: the attribute value if found, null otherwise

---


### afterInvocation

```
public void afterInvocation(IInvokedMethod method,
                            ITestResult testResult)
```

:   **Specified by:**: `afterInvocation` in interface `IInvokedMethodListener`

---


### beforeInvocation

```
public void beforeInvocation(IInvokedMethod method,
                             ITestResult testResult)
```

:   **Specified by:**: `beforeInvocation` in interface `IInvokedMethodListener`

---


### getAllInvokedMethods

```
public java.util.List<IInvokedMethod> getAllInvokedMethods()
```

:   **Specified by:**: `getAllInvokedMethods` in interface `ISuite`
:   **Returns:**: a list of all the methods that were invoked in this suite.

---


### getAllMethods

```
public java.util.List<ITestNGMethod> getAllMethods()
```

:   **Specified by:**: `getAllMethods` in interface `ISuite`
:   **Returns:**: the total number of methods found in this suite. The presence of factories or data providers might cause the actual number of test methods run be bigger than this list.


---


|  |  |  |
| --- | --- | --- |
| **Overview** \| **Package** \| **Class** \| **Tree** \| **Deprecated** \| **Index** \| **Help** | |  |
| **PREV CLASS**   **NEXT CLASS** | **FRAMES**    **NO FRAMES**     **All Classes** |  |
| SUMMARY: NESTED \| FIELD \| CONSTR \| METHOD | DETAIL: FIELD \| CONSTR \| METHOD |  |


---
